# A mobile app as support for pelvic floor muscle training started prior to radical prostatectomy

**DOI:** 10.1002/bco2.142

**Published:** 2022-03-09

**Authors:** Zinah Al‐Zaidi, Anna Lindam, Per Fransson, Eva Samuelsson

**Affiliations:** ^1^ Family Medicine, Department of Public Health and Clinical Medicine Umeå University Umeå Sweden; ^2^ Unit of Research, Education, and Development, Östersund Hospital, Department of Public Health and Clinical Medicine Umeå University Umeå Sweden; ^3^ Department of Nursing Umeå University Umeå Sweden

**Keywords:** mobile application, pelvic floor muscle training, radical prostatectomy, self‐management, urinary incontinence

## Abstract

**Objective:**

To evaluate the usefulness of a mobile app to support pelvic floor muscle training (PFMT) started prior to radical prostatectomy (RP).

**Materials and methods:**

A prospective cohort study conducted in Sweden from June 2018 to February 2021 including men for whom RP was planned within 12 months. Users responded anonymously to questionnaires at baseline, 1 and 3 months. Our primary aim was to evaluate if the app could facilitate PFMT and increase confidence in performing pelvic floor muscle (PFM) contractions correctly. Our second aim was to describe the change in urinary incontinence (UI) after RP, based on the International Consultation on Incontinence Questionnaire‐Urinary Incontinence Short Form (ICIQ‐UI SF).

**Results:**

Of the 3043 users at baseline, 388 met the primary inclusion criteria. Of those, 71 (18.3%) were incontinent, predominantly with slight symptoms. The most common type was urge UI, 39/71 (54.9%). Of the 388 users, 159 (41.0%) answered the questionnaire at 1 month, and 131 (33.7%) at 3 months within 89–135 days. Of those 131, 127 (96.9%) indicated that the app facilitated their training ‘a lot’ or ‘somewhat’. Confidence in performing PFM contractions correctly increased from 39.7% at baseline to 74.0% at 1 month and 87.8% at 3 months (*p* < 0.001). At baseline, 19.8% performed PFM contractions at least daily, which increased to 74.0% at 1 month and 77.9% at 3 months (*p* < 0.001). At 3 months, 115/131 (87.8%) had undergone RP, 93.6% of which were robot‐assisted. Of the 115, 103 (89.6%) were incontinent, and stress UI dominated. The mean ICIQ‐UI SF score increased from 1.2 (2.4 SD) at baseline to 9.6 (5.2 SD), *p* < 0.001, after surgery.

**Conclusions:**

The mobile app facilitated pelvic floor muscle training for men who were planned to undergo radical prostatectomy and used the app.

## 1 INTRODUCTION

Prostate cancer is one of the most common types of cancer in the world. In 2020, it had the fourth highest incidence rate of all cancers globally.^1^ The most common treatment options of the disease are radical prostatectomy (RP) and radiotherapy. Urinary incontinence (UI) affects most patients directly after RP, and 18% of patients report having severe or very severe leakage 1 year after undergoing robot‐assisted RP in Sweden, whereas 27% experience severe leakage after radical retropubic prostatectomy (RRP).^2^

Pelvic floor muscle training (PFMT) is the recommended first‐line treatment for prostatectomy‐related UI, and both pre‐operative and post‐operative PFMT are recommended in national healthcare guidelines in many countries including Sweden.^3^ The evidence regarding the efficacy of pre‐operative PFMT has been much debated during the past decade. Evidence in favour of PFMT being implemented prior to RP has been provided by many studies, including two systematic reviews and meta‐analyses.^4^, ^5^, ^6^ Furthermore, the systematic review from 2020 demonstrated that between pre‐operative and post‐operative PFMT, only pre‐operative training was associated with reduced incontinence.^5^ Meanwhile, a systematic review conducted in 2020 suggested that there was a lack of evidence to support the effectiveness of both pre‐operative and post‐operative PFMT, although it was stated that further high‐quality studies were needed in order to confirm those findings.^7^ However, a recent study published in April 2021 that pooled evidence from several studies concluded that pre‐operative PFMT was more effective in terms of the faster return of continence after RP than post‐operative training alone.^8^

In conjunction with the ongoing COVID pandemic, demand for and interest in e‐health has increased remarkably, especially in urology.^9^ The mobile application Tät®, intended for the self‐treatment of stress UI in women, has been evaluated in an RCT in which efficacy was demonstrated in terms of improvement in incontinence symptoms, leakage and quality of life.^10^ The effectiveness of this app has also been shown in a large ‘real‐world’ population.^11^

The Tät® III app was developed as a result of requests from men undergoing RP who wanted support with PFMT. In a previous pilot feasibility study of this app (presented as an abstract at the 2019 International Continence Society Congress in Gothenburg), most of the men who participated had already undergone RP, and one third of them had undergone surgery more than 1 year prior to downloading the app. As the target group for the Tät III app is mainly men planned to undergo RP, we wanted to conduct a larger study of this population aimed at evaluating whether the app could facilitate PFMT and increase the level of confidence in performing pelvic floor muscle contractions correctly. Our second aim was to describe the characteristics of the users, the percentage of users who continued training with the support of the app after 3 months and the change in incontinence symptoms after surgery.

## 2 SUBJECTS AND METHODS

### 2.1 Study design

This prospective cohort study conducted from June 2018 to February 2021 was based on data collected from questionnaires answered by users of the Tät III app within this time period. The app was freely available via Google Play and App Store. Upon download, the users were informed about the study and invited to answer questionnaires anonymously on three different occasions: at download, after 1 month and at 3 months after download. The users were able to use the full version of the app even without responding to the questionnaires. The 1‐month follow‐up became available to the users 30 days after download, but the users had the option to postpone answering it. If the 1‐month follow‐up was not answered within 90 days, the 3‐month follow‐up was still made available to the users.

The anonymous data from the questionnaires were linked to a unique app‐ID and transferred as encrypted data to a secure database at Umeå University. No data were stored in the app. In the database, the three different questionnaires could be merged via the app‐ID. The answers could not be traced back to the user, and no information about their name, social security number, e‐mail, telephone number, IMEI code or IP address was asked for. As such, no personal data were collected for this study.

### 2.2 The Tät III app

The Tät III app was based on the same structure as Tät for women. It was developed by Eva Samuelsson in collaboration with software technicians at Umeå university. Texts were written with input from urotherapists and a urologist. The app was initially tested by a group of men that had undergone RP and was evaluated in a small pilot study. A new updated version of the app with minor text changes and a new function that allowed the addition of sound to the exercises was later released in December 2019. Another updated version of the app, now also available in English, was subsequently released in April 2020. Both prior to and during the study, the app was tested by family doctors, urotherapists and users. Moreover, the users were able to contact the research team by using a contact e‐mail on our web site, as well as adding free‐text answers in the questionnaires for feedback and further views.

**FIGURE 1:**
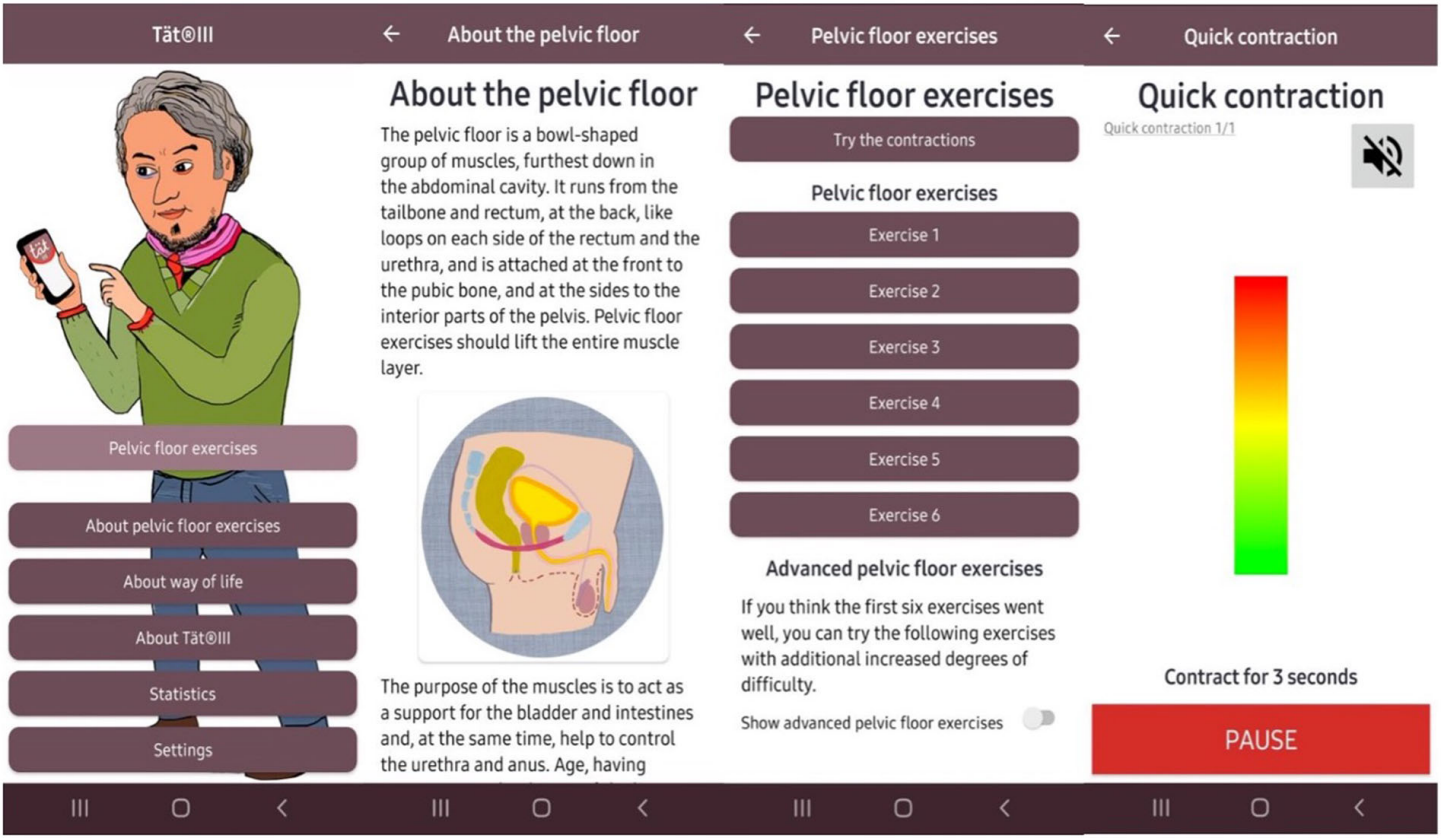
Screenshots from the Tät III app

The Tät III app is intended to support men who are planned to undergo RP to perform PFMT, and it is currently available in both Swedish and English. The app provides 12 training stages, six with basic exercises and six with advanced exercises of different levels and increased difficulties. All exercises are available with visual and audio instructions and graphical features that illustrate the duration and intensity needed to perform a pelvic muscle contraction correctly (Figure 1).

The app also provides a statistical function to enable users to track their training as well as a function for setting reminders for training. Moreover, the users have access to other services such as information about lifestyle habits, PFMT and prostate cancer surgery.

### 2.3 Study participants

This study included men who answered the baseline questionnaire between 28 June 2018 and 27 September 2020 and indicated that they were planned to undergo a prostatectomy within 12 months. Users who had already undergone RP or transurethral resection of the prostate (TURP), researchers and test users were excluded. Baseline characteristics were analysed for those included at baseline.

The 1‐month questionnaires were answered between 28 July 2018 and 8 December 2020, and the 3‐month questionnaires were answered between 27 September 2018 and 8 February 2021. The follow‐up questionnaires were merged with the baseline questionnaires using the unique app‐ID given to every user.

In the follow‐up analyses, we included participants that responded to the 3‐month questionnaires within 89–135 days after the baseline questionnaire. This group was considered to have continued training with the support of the app for at least 3 months, and the reason for choosing the time limit of 89–135 days was to ensure that active app users were included. In this group, we analysed user evaluation, confidence in performing pelvic floor muscle contractions correctly and PFMT frequency.

**FIGURE 2:**
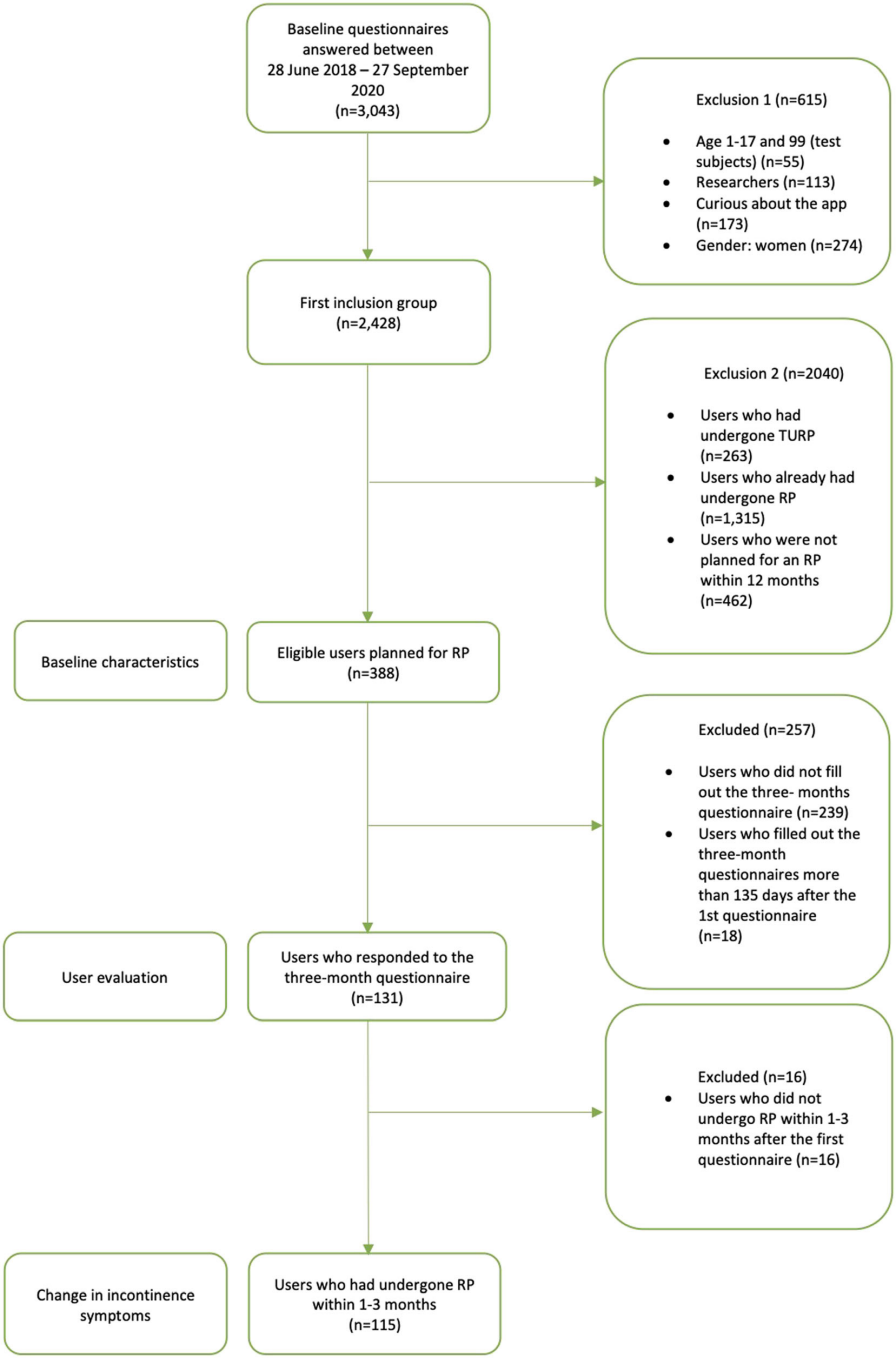
Flowchart of the users of the Tät III app at baseline and at 3‐month follow‐up

We analysed changes in incontinence symptoms in the group that answered the 3‐month follow‐up questionnaires within 89–135 days after the first questionnaire and had undergone RP within 1–3 months after the first questionnaire (Figure 2).

### 2.4 The questionnaires

The baseline questionnaire included demographic questions such as age, country, level of education, reason for download and previous TURP or RP. Additionally, it included a question on PFMT frequency on a scale from ‘never’, ‘less than once a week’, ‘1‐6 times a week’, ‘daily’ to ‘3 times a day or more often’. There was also a ‘yes’ or ‘no’ question on whether the users felt confident in their ability to identify pelvic floor muscles and perform muscle contractions correctly. The users were also asked whether they had received information about PFMT from medical professionals.

Incontinence symptom severity for each user was calculated using the ‘International Consultation on Incontinence Questionnaire‐Urinary Incontinence Short Form’ (ICIQ‐UI SF),^12^ which is based on three self‐diagnostic questions regarding frequency, amount of leakage and overall impact on daily life. The users are given a certain number of points for each question depending on how they answer. The number of points for all three questions is totaled to get a score on a scale from 0 to 21, with higher scores indicating more severe symptoms. The total scores are then categorised into four severity groups defined by Klovning et al.^13^: slight,^1^, ^2^, ^3^, ^4^, ^5^ moderate,^6^, ^7^, ^8^, ^9^, ^10^, ^11^, ^12^ severe^13^, ^14^, ^15^, ^16^, ^17^, ^18^ and very severe.^19^, ^20^, ^21^

In this study, users were considered ‘incontinent’ if they indicated that they had both some amount of leakage and some leakage frequency. The type of incontinence was categorised into four categories based on the ICIQ‐UI SF, using a method from a study of women with incontinence by Espuña‐Pons et al.^14^: urgency urinary incontinence (UUI), stress urinary incontinence (SUI), mixed urinary incontinence (MUI) and other types. Users were considered to have SUI if they indicated having leakage when coughing or sneezing and/or when exercising/physically active and did not leak before reaching the toilet. Those who had leakage before reaching the toilet but not while physically active, coughing or sneezing were considered to have UUI. Users who had leakage both before reaching the toilet and while physically active, coughing and/or sneezing were considered to have MUI. Lastly, those who indicated leakage at other times than those mentioned above, such as while sleeping, after urinating, leakage for no apparent reason or constant leakage, were classified as ‘other’.

The 1‐ and 3‐month questionnaires included follow‐up questions regarding participants' confidence in their ability to perform muscle contractions correctly, training frequency and ICIQ‐UI SF scores. They also included additional questions regarding user satisfaction and views on the app. Lastly, the users were asked to answer the question ‘Does the app facilitate training?’, which had the answer options ‘Yes, a lot’, ‘Yes, somewhat’, ‘No, not really’ and “No, not at all”.

### 2.5 Statistical analyses

Descriptive characteristics of the users at baseline were reported using means and percentages. The differences in the characteristics between users who continued training with the support of the app for at least 3 months and those who did not were calculated. We used *T* tests to compare means such as ICIQ‐UI scores, Mann–Whitney's test for median age and chi‐square tests were used for categorical variables. Potential differences in education level were analysed using logistic regression to adjust for age, which was believed to be a confounding factor in this study population.

Differences in user evaluation of the app at 1 and 3 months were determined using Wilcoxon's Signed Rank test. Furthermore, the differences in app‐usage frequencies and ability to perform muscle contractions correctly at baseline, 1 and 3 months were measured using the Friedman test and Cochran's *Q* test. Chi‐square test and Fisher's exact test were used to determine whether there was a difference between those who had received information about PFMT and those who had not, in terms of confidence in their ability to perform PFM contractions correctly and whether the app facilitated their training.

For the changes in symptoms in users who underwent RP within 1–3 months after the first questionnaire, a paired *t* test was used to analyse ICIQ‐UI SF score differences, and Wilcoxon's Signed Rank test was used to analyse differences in severity and types of incontinence between those factors at baseline and after 3 months. All of the tests mentioned above were conducted using the IBM SPSS statistical software version 27.

### 2.6 Ethics and data protection

The Tät III app was CE‐marked as a medical device product class I in accordance with regulations for medical technology ([EU] 2017/745 MDR). This study was approved by the Regional Ethical Review Board, Umeå with a diary number of Dnr: 2018/160‐31. An advisory opinion only was given because no personal data were collected for this study. Before answering the questionnaires, the users had to tick a box to confirm that they had read the information about the questionnaire, which was a measure taken to ensure that the users were well informed about the study.

## 3 RESULTS

The baseline questionnaire was answered by 3043 users during the period of 28 June 2018 to 27 September 2020. Of those, 388 (12.7%) users met the initial inclusion criteria. Of those users, 159 (41.0%) answered the 1‐month questionnaire, and 131 (33.7%) answered the 3‐month follow‐up questionnaire within 89–135 days. Of the 131 users, 100 (76.3%) had also answered the 1‐month follow‐up questionnaire. During the 3‐month study, RP had been performed on 115 of these 131 men (87.8%).

### 3.1 Characteristics of the users at baseline

The absolute majority of the users were from Sweden, most of them lived in a city with between 50 000 and 1 000 000 inhabitants, and the majority indicated ‘university’ as their highest level of education. The question regarding reason for download was a multiple‐response question, and the most common reason for download was for ‘preventive training’ 376/388 (96.9%) followed by training to ‘improve erectile function’ 87/388 (22.4%), ‘training to improve incontinence’ 48/388 (12.4%) and ‘training for other reasons’ 16/388 (4.1%) (Table 1).

**TABLE 1:** Characteristics of the users at baseline

	Eligible users planned for RP (*n* = 388)	Users who responded to 3‐month follow‐up (*n* = 131)	Users who did not respond to 3‐month follow‐up (*n* = 257)	Difference between group who responded to 3‐month follow‐up and those who did not, *p* value
Age, median (quartiles)	65.0 (61; 69)	65.0 (61; 69)	64.0 (60; 69)	0.170^a^
Language, *n* (%)				0.975^b^
Swedish	376 (96.9)	127 (96.9)	249 (96.9)	
Other	12 (3.1)	4 (3.1)	8 (3.1)	
Country, *n* (%)				0.593^b^
Sweden	372 (95.9)	127 (96.9)	245 (95.3)	
Other	16 (4.1)	4 (3.1)	12 (4.7)	
Highest level of education, *n* (%)				0.071^c^
0–9 years of school	45 (11.6)	11 (8.4)	34 (13.2)	
9–12 years of school	158 (40.7)	49 (37.4)	109 (42.4)	
University	185 (47.7)	71 (54.2)	114 (44.4)	
Place of residence, *n* (%)				0.951^b^
Rural area	64 (16.5)	20 (15.3)	44 (17.0)	
Place/town <50 000 people	115 (29.6)	38 (29.0)	77 (30.0)	
Town/city 50 000–1 000 000 people	147 (37.9)	51 (38.9)	96 (37.4)	
Major city >1 000 000 people	62 (16.0)	22 (16.8)	40 (15.6)	
Incontinent users, *n* (%)	71 (18.3)	20 (15.3)	51 (20.0)	0.270^b^
Mean ICIQ‐UI SF^d^ of incontinent users (SD)	5.18 (1.9)	4.75 (1.8)	5.35 (1.9)	0.233^e^
Symptom severity of incontinent users, *n* (%)				0.597^b^
Slight	50 (70.4)	15 (75.0)	35 (68.6)	
Moderate or higher	21 (29.6)	5 (25.0)	16 (31.4)	

Abbreviations: ICIQ‐UI SF, International Consultation on Incontinence Questionnaire‐Urinary Incontinence Short Form; RP, radical prostatectomy.

^a^ Mann–Whitney's test.

^b^ *χ* ^2^ test.

^c^ Logistic regression adjusted for age.

^d^ ICIQ‐SF score based on International Consultation on Incontinence Questionnaire‐Urinary Incontinence Short Form (ICIQ‐UI SF).

^e^ *t* test.

Regarding whether the users had received information about PFMT from medical professionals, 98/388 (25.2%) answered ‘no’, 112/388 (28.9%) answered ‘yes, but insufficient’ and 178/388 (45.9%) indicated that they had received sufficient information.

Of the 388 users, 71 (18.3%) were considered incontinent. Of those, 39/71 (54.9%) were categorised as urgency UI and 7/71 (9.9%) as stress UI, based on the symptoms reported. The majority (70.4%) had slight symptoms of incontinence, whereas the remaining 29.6% had moderate symptoms. No user suffered from severe or very severe symptoms (Table 1).

### 3.2 Characteristics of users who continued training with the support of the app up to 3 months

Of the 388 users at inclusion, 131 (33.7%) answered the 3‐month follow‐up questionnaire within 89–135 days. There were no statistically significant differences in the characteristics between those who responded to the questionnaire at 3 months and continued training with support of the app and those who did not. We adjusted for age when we compared the level of education between the groups. After adjustment, no differences in education levels were seen (Table 1).

### 3.3 User‐evaluation and subjective change in ability to perform pelvic muscle contractions correctly

The app facilitated training for the users who continued training for 3 months, 95/100 (95.0%) indicated that the app facilitated their training ‘a lot’ or ‘somewhat’ at the 1‐month follow‐up, and the percentage increased to 127/131 (96.9%) at the 3‐month follow‐up. Among those who had received information about PFMT before the study started, 95/96 (98.9%) stated that the app facilitated training at 3 months. This corresponds to 32/35 (91.4%) in the group who had not received information, and no significant difference was found (*p* = 0.058).

There were significant differences in the proportion of participants that felt confident in performing muscle contractions correctly between the three timepoints; the users felt more confident with each follow‐up, and the percentage of confident users reached 87.8% at 3 months. The users also had notably high training frequencies, with 74.0% and 77.9% training at least daily at 1 and 3 months, respectively (Table 2). At baseline, only 6/35 (17.1%) of those who had not received information about PFMT felt confident in their ability to perform PFM contractions correctly, whereas 12/34 (35.3%) of those who had received some information, and 34/62 (54.8%) of those who had received more extensive information felt confident in their ability. The difference between groups was significant at baseline (*p* = 0.001). At 3 months, the percentage of users that felt confident in performing PFM contractions correctly was similar regardless of whether they had got information before the study or not, 29/35 (82.9%) without information, 29/34 (85.3%) with some information and 57/62 (91.9%) with sufficient information (*p* = 0.335).

**TABLE 2:** Confidence in ability to perform pelvic muscle contractions correctly and training frequency at baseline, 1 and 3 months

	At baseline (*n* = 131)	1 month (*n* = 100)	3 month (*n* = 131)	*p* value
Confident in ability to perform muscle contractions correctly? *n* (%)				<0.001^a^
Yes	52 (39.7)	74 (74.0)	115 (87.8)	
No	79 (60.3)	26 (26.0)	16 (12.2)	
PFMT^b^ training consistency for the past 4 weeks? *n* (%)				<0.001^c^
Never	62 (47.3)	7 (7.0)	2 (1.5)	
Less than once a week	20 (15.3)	3 (3.0)	5 (3.8)	
1–6 times/week	23 (17.6)	16 (16.0)	22 (16.8)	
Daily	18 (13.7)	43 (43.0)	63 (48.1)	
3 times/day or more often	8 (6.1)	31 (31.0)	39 (29.8)	

^a^ Cochran *Q* test.

^b^ PFMT—pelvic floor muscle training.

^c^ Friedman test.

### 3.4 ICIQ scores and changes in symptoms

Of the 115 men that had undergone RP within 1–3 months after the first questionnaire, 19 (16.5%) were incontinent before the RP at baseline, which increased to 103 (89.6%) at the 3‐month follow‐up. The mean ICIQ‐UI SF increased from 1.17 (2.4 SD) before RP to 9.58 (5.2 SD) after RP (*p* < 0.001). The most common type of incontinence at baseline was urgency UI, and the shift to stress and mixed UI as well as the increased severity of symptoms was evident after RP (Table 3).

**TABLE 3:** Change in incontinence symptoms in users who had undergone radical prostatectomy within 1–3 months

	At baseline (*n* = 115)	3‐month follow‐up (*n* = 115)	*p* value
Mean ICIQ‐UI SF^a^ score all users (SD)	1.17 (2.4)	9.58 (5.2)	<0.001^b^
Incontinent users, *n* (%)	19 (16.5)	103 (89.6)	<0.001^c^
Type of incontinence all users, *n* (%)			<0.001^d^
Stress urinary incontinence	2 (1.7)	60 (52.2)	
Urgency urinary incontinence	13 (11.3)	9 (7.8)	
Mixed urinary incontinence	1 (0.9)	33 (28.7)	
Other types of leakage	7 (6.1)	7 (6.1)	
None	92 (80.0)	6 (5.2)	
Severity of incontinence all users, *n* (%)			<0.001^d^
None	88 (76.5)	6 (5.2)	
Slight	19 (16.5)	18 (15.7)	
Moderate	8 (7.0)	54 (46.9)	
Severe	0 (0)	33 (28.7)	
Very severe	0 (0)	4 (3.5)	

^a^ ICIQ‐UI SF, International Consultation on Incontinence Questionnaire‐Urinary Incontinence Short Form.

^b^ Paired *t* test.

^c^ MC Nemar's test.

^d^ Wilcoxon's Signed Rank test.

## 4 DISCUSSION

The results of this study show that approximately one third of the men who were planned for an RP and downloaded the app to support PFMT continued using the app for 3 months. Nearly all of those indicated that the app facilitated their training. Their confidence in performing pelvic muscle contractions correctly and the training frequency increased during the 3‐month period. At the 3‐month follow‐up and after undergoing RP, the number of incontinent users increased, and the predominant type of incontinence shifted from UUI to mostly SUI or MUI, with considerably higher severity.

As far as we are aware, this is the first study that evaluates an app aimed to support pelvic muscle training in men undergoing RP. One previous study from 2015 showed that an app intended to support self‐management of UI was a useful tool for documenting incontinence and improvement in the men and women included.^15^

This study demonstrated that the users who continued training for 3 months increased their training frequency, and more than 70% trained daily or more often at 3 months. This indicates a very high level of adherence, which is important both for the short‐term and long‐term effects of PFMT.^16^ For comparison, in a real‐world study of the effectiveness of the Tät app for women, 28.9% of the users trained daily or more often at 3 months.^11^ The group of men that are planned to undergo RP seems to be highly motivated to do PFMT.

Apps have the ability to improve adherence for treatment in chronic diseases,^17^ and the difficulty in remembering to exercise is a common factor that reduces adherence in PFMT.^18^ The Tät III app offers features that motivate users to do PFMT and also has reminder functions. These factors were appreciated by women in previous studies.^19^

Our results also showed that confidence in performing contractions correctly increased at each follow‐up. This finding was based on responses from the questionnaires, and confidence was self‐assessed. The Tät III app provides detailed instructive advice to guide users to perform pelvic contractions correctly, and it has been shown that most women are able to learn how to perform pelvic contractions correctly without clinical supervision.^20^ In women with stress UI, there is no strong evidence that supervised PFMT is more effective than unsupervised PFMT.^21^, ^22^, ^23^, ^24^ A previous study of men undergoing RP showed that there was no difference between written PFMT guidance and verbal PFMT guidance via telephone regarding effectiveness.^25^

In this study, the ICIQ‐UI SF scores were calculated to show the change in symptoms after RP, and there was no intention to use the scores as a measure of effectiveness of the app. The preoperative score of 1.2 in our study is consistent with the preoperative score in a recent large‐scale study.^26^ However, the 3‐month scores were higher in our study, which could be due to the fact that the timing between surgery and follow‐up was not homogenous.

One of the limitations of this cohort study was the loss to follow‐up. However, there were no significant differences in baseline characteristics between those who continued their training and those who did not. Another limitation was that the questionnaires were anonymous, which meant that we could not contact the participants to verify the type and severity of incontinence nor whether the exercises were performed correctly.

Almost all participants at 3 months stated that the app facilitated their training. This finding may be partly explained by follow‐up bias, because those who were satisfied with the app at the first follow‐up might have been more motivated and thus more likely to continue using it for at least 3 months. Also, we cannot be certain that the high training frequency of the users at 3 months was solely a consequence of using the app. Moreover, according to the Swedish national cancer strategy,^3^ all patients are offered their own permanent contact‐nurse who can also provide information about PFMT and, if necessary, offer to put the patient in contact with a physiotherapist. In this study, most users had received information about PFMT, but we do not know if the users received further guidance regarding PFMT from their clinic during the study, which could constitute another selection bias.

One strength of this study is the real‐world setting, with the inclusion of a relatively large and clinically relevant group of men. Another strength is that there were no technical difficulties with the app or the questionnaires during the study. Furthermore, we used a recommended and validated score for the severity of incontinence, and we had no internal data loss because it was mandatory to answer all the questions in the questionnaires.

The men in our study seem to be representative of the general Swedish population of men that undergo RP. In our study, the median age was 65 years, and 89.1% had undergone a robot‐assisted RP. This corresponds well with the median age of 66 years and 93.6% robot‐assisted operations per the National Prostate Cancer Registry (NPCR) based on 2771 RPs during 2020.^27^ In our study, the prevalence of incontinence was 16.5% before RP; the corresponding register data based on questionnaires showed that 21.7% stated that they had little, moderate or much leakage; and according to another question regarding the amount of leakage, 11.8% stated that they had urinary leakage before RP.

In this study, we included the 388 men that were planned to undergo RP and that downloaded the app. Additionally, 1315 users had already had an RP when they downloaded the app. Within the studied period of 27 months, approximately 6881 RPs were performed in Sweden. Apparently, the app was downloaded by a considerable portion of men undergoing RP, which indicates that apps are also appreciated for support of PFMT in this group.

## 5 CONCLUSION

A mobile app designed to support PFMT that is started prior to RP was appreciated by the users and facilitated their training. The app can both be used by individuals as a tool to support training and potentially serve as an additional option for the clinic to enhance knowledge about PFMT. However, controlled studies are needed to evaluate the efficacy of the app for the prevention and treatment of UI after RP.

## CONFLICT OF INTEREST

The logos Tät and Tät.nu are registered as trademarks by the Swedish Patent and Registration office for eContinence AB, a Swedish e‐health company founded in July 2021 with the aim of maintaining, distributing, commercialising and further developing the apps created within the research project Tät.nu (eContinence.se). Eva Samuelsson is a co‐founder and shareholder of eContinence AB and the Managing Director. None of the other authors declare any conflict of interest.

## AUTHOR CONTRIBUTIONS

ES designed and led the study at all stages. ZA performed the statistical analyses of the trial data, and ES and AL participated in the analyses. PF extracted data from the NPCR. All authors participated in the interpretation of the data. ZA wrote the manuscript, with substantial input from ES. All authors critically reviewed successive drafts of the report and approved the final version of the manuscript before publication.
